# Pertinence of Salt-Related Knowledge and Reported Behaviour on Salt Intake in Adults: A Cross-Sectional Study

**DOI:** 10.3390/nu15194114

**Published:** 2023-09-23

**Authors:** Georgios Marakis, Ana Marques Domingues, Anna Crispo, Emmanuella Magriplis, Eleni Vasara, Lamprini Kontopoulou, Christos Triantafyllou, Petros Skepastianos, Sousana K. Papadopoulou, Nikolaos E. Rodopaios, Maria Hassapidou, Antonis Zampelas, Francesco P. Cappuccio, João Breda

**Affiliations:** 1 Hellenic Food Authority, 124 Kifisias Av. & 2 Iatridou Str., 11526 Athens, Greece; gmarakis@efet.gr (G.M.); azampelas@aua.gr (A.Z.); 2 WHO Athens Quality of Care and Patient Safety Office, Ploutarchou 3, 10675 Athens, Greece; jesusa@who.int (A.M.D.); a.crispo@istitutotumori.na.it (A.C.); triantafyllouc@who.int (C.T.); 3 Istituto Nazionale dei “Tumori Fondazione G. Pascale”, Via Mariano Semmola 52, 80131 Napoli, Italy; 4 Department of Food Science and Human Nutrition, Agricultural University of Athens, Iera Odos 75, 11855 Athens, Greece; emagriplis@aua.gr; 5 School of Biology, Aristotle University of Thessaloniki, University Campus, 54124 Thessaloniki, Greece; evasara@bio.auth.gr; 6 Department of Nursing, University of Thessaly, Gaiopolis Campus, Larissa-Trikala Ring-Road, 41500 Larissa, Greece; lamprini2@yahoo.gr; 7 Department of Medical Laboratory Studies, International Hellenic University, 57400 Thessaloniki, Greece; pskep@otenet.gr; 8 Department of Nutritional Sciences and Dietetics, International Hellenic University, 57400 Thessaloniki, Greece; souzpapa@gmail.com (S.K.P.); mnhas@ihu.gr (M.H.); 9 Medical School, University of Crete, 71003 Heraklion, Crete, Greece; nikow1966@yahoo.gr; 10 Medical School, University of Warwick, Coventry CV4 7AL, UK; f.p.cappuccio@warwick.ac.uk; 11 WHO Collaborating Centre for Nutrition, Warwick Medical School, Coventry CV4 7AL, UK

**Keywords:** knowledge, behaviour, 24 h urinary sodium excretion, salt reduction

## Abstract

The association between salt-related knowledge, attitude, behaviour (KAB) and actual salt consumption in Greek adults is uncertain. This study investigates the correlation between salt intake, gauged by 24-h urinary sodium excretion, with salt-related KAB. It further explores how socio-demographic factors influence these behaviors. Salt consumption was evaluated using a 24-h urinary sodium test, and compared to self-reported KAB data. Knowledge and behavior scores related to salt were computed. An overall cohort-adjusted model examined the relationship between daily salt consumption, knowledge and behavior scores, and certain covariates. Through the stratification by the cohort random effect, two models were established (Cohort I Adults; Cohort II Students) examining the same relationships of the overall cohort model. 463 Greek adults participated. The average salt intake was 9.54 g/day, nearly double the WHO recommendation. Significant differences in knowledge scores were noted based on sex, age, education, and BMI. A trend suggesting lower discretionary salt use with increased salt intake was observed (*p* = 0.06). However, comprehensive analysis revealed no direct correlation between salt intake and either knowledge (*p* = 0.562) or behavior scores (*p* = 0.210). The results emphasize the need for food product reforms by industry stakeholders and accelerated efforts towards reducing salt intake.

## 1. Introduction

The overconsumption of salt is a well-established and significant determinant of blood pressure (BP), accounting for approximately 30% of hypertension prevalence worldwide [1]. Regardless of BP status, the evidence suggests an approximately linear association between dietary sodium intake and a decrease in both systolic and diastolic BP [2]. In the World Health Organization (WHO) Global Action Plan for the Prevention and Control of Noncommunicable Diseases (NCDs), the WHO Member States have agreed on the voluntary global target of a 30% relative reduction in mean population salt intake by 2025 [3]. In fact, even a modest reduction in salt intake can have beneficial effects on BP and considerable health benefits at the population level despite hypertensive status, sex, and ethnic group [4], and indirectly reduce overall mortality at a low cost [5].

The latest census in Greece has provided evidence of an increasing death rate from circulatory system diseases, rising from 10.0% in 1938 to 34.9% in 2020 [6]. The prevalence of hypertension among Greek adults is high [7,8] and varies significantly across different age groups within each sex [7]; furthermore, high blood pressure has also been reported among Greek children [9]. Regarding salt intake, both adults [10,11] and children [7] have a salt consumption well above the no more than 5 g of salt per day recommendation by WHO [12]. High blood pressure levels have also been reported in children [9]. However, data on salt intake estimated with the use of 24 h urine collection from a nationally representative sample in Greece is still lacking. The Hellenic National Nutrition and Health Survey (HNNHS) has assessed the contribution of salt from processed foods alone to total salt intake and found that the cereals group (processed cereals and bread) is a major driver of overall salt intake [11]. The contribution of discretionary salt to overall intake has not been estimated in Greece yet.

The Greek Salt Reduction Strategy, in line with the general EU strategy [13], has identified consumer education and awareness campaigns on salt intake and its implications as a key pillar. However, based on the latest available scientific evidence and in accordance with the country’s actual situation [14], it is imperative to prioritize specific salt-related initiatives to insure a cost-effective use of resources. While acknowledging the importance of consumers’ awareness, it is unclear to what extent salt-related knowledge and attitude can actually lead to a decrease in salt intake. In the review by Spronk et al. [15] on nutrition knowledge and dietary intake, most studies found some positive relationship between self-reported knowledge and dietary intake, which was later confirmed by more recent studies [16,17]. On the contrary, other research suggests that measured salt intake is inconsistent with people’s knowledge, attitude, and reported salt-related knowledge, attitude, and behaviour (KAB). Several scientific studies have focused on either salt intake or salt-related KAB (e.g., [18]), however, fewer have explored the association between these variables.

Therefore, the objective of this study was to evaluate the association between salt intake, assessed by 24 h urinary sodium excretion, and salt-related KAB among adults in central and northern Greece, and to analyze variations in salt-related KAB based on different socio-demographic characteristics.

## 2. Materials and Methods

### 2.1. Description of the Study—Study Population

The present used cross-sectional data on salt-related knowledge, attitude and be-haviour, and salt intake among adults in central and northern Greece. The dataset combined information from two cohorts: the first cohort encompassed the general pop-ulation in northern Greece, specifically the Thessaloniki greater metropolitan area (the salt intake data but not the KAB data from this cohort has been previously published [10]), while the second cohort comprised undergraduate students from the Aristotle University of Thessaloniki (Thessaloniki, Greece), the Alexander Technological and Educational Institute of Thessaloniki (Thessaloniki, Greece) and the Technological and Educational Institute of Thessaly (Karditsa, Greece). The same study protocol was applied to both cohorts to ensure uniformity. Data collection for the first cohort occurred from February 2015 to March 2016 and for the second cohort between April 2016 and February 2017. Notably, no urinary samples were collected during festive seasons; however, samples could be obtained on both working days and weekends. 

Recruitment of volunteers in the first cohort was carried out at various sites and venues [10], whereas in the second cohort, recruitment was done exclusively at the premises of higher educational institutes/universities. In the first cohort, 252 individuals were included in the analyses (of which 52 were students from the Aristotle University of Thessaloniki). In the second cohort, following the same strict quality control procedures, 211 participants were eligible to be included in the analysis (a. Aristotle University of Thessaloniki (n = 81), b. International Hellenic University (n = 43) and c. the University of Thessaly (n = 87)). The total number of students in both cohorts was 263. 

Pregnant and lactating women as well as individuals with a medical diagnosis of hypertension (whether on an anti-hypertensive treatment or not), diabetes mellitus as well as those with heart, liver, renal, gastrointestinal, or neoplastic diseases were excluded from the study.

Participation in this study was voluntary and anonymous. Participants were free to withdraw at any time. All participants provided informed consent and ethics approval was granted by the Ethics Committee of the Alexander Technological and Educational Institute of Thessaloniki [10] which is now merged with the International Hellenic University. No financial incentive was offered to participants.

### 2.2. Anthropometric Data and Salt Intake Estimation

Height and weight were measured in subjects wearing lightweight clothing and without shoes. Body weight measurements were obtained using a Tanita BWB-800S digital scale, and body height was measured using a stable stadiometer. Body mass index (BMI) was calculated as weight (kg)/height^2^ (m^2^). Waist circumference (cm) was measured around the midpoint between the costal margin and the iliac crest during expiration.

Salt intake in both cohorts followed exactly the same strict protocol [10] and validation criteria to avoid over and under urinary collections. Participants were provided with required equipment, along with verbal and written instructions, to facilitate the collection of the single 24-h urine sample. Urine collections were rejected if the participant admitted that a sample was missed from the collection or if the timing of the collection fell outside the range of 23–25 h. Urine collections were also suspected to be inaccurate if urinary volumes were <500 mL or if urinary creatinine (UCr) was less than 2 standard deviations from the sex-specific mean.

For each individual, the 24-h sodium excretion value was calculated as the concentration of sodium in the urine (mmol/L) multiplied by the urinary volume (L/day). In order to convert the urinary output to dietary intake, the urinary excretion of sodium values (mEq/day) was first converted to mg/day. Then, sodium values were multiplied by 1.05 to estimate sodium intake to allow for non-urinary sodium losses, as previously described [10].

Urine analyses in both cohorts were performed in the same lab by the same investigator. The urine volume of the 24-h collection was measured and a 10 mL aliquot was stored at −20 °C until analysis. Urinary sodium excretions were determined by ion-selective electrode potentiometry (ATVIA 1800 Siemens, ISE buffer Siemens AG, Munich, Germany) and by taking into account the exact 24-h adjusted urinary volume. Creatinine was measured using the Jaffe method (ATVIA 1800 Siemens AG, Munich, Germany).

### 2.3. Assessment of Knowledge, Attitude, and Behaviour Related to Salt 

On the day the investigators collected 24-h urine samples, participants were asked to fill in a self-administered questionnaire regarding salt-related KAB, previously developed by the Hellenic Food Authority and used in a nationally representative sample of Greek adults in 2011 (Questionnaire) [19]. The original questionnaire included seventeen pre-coded questions. However, the statistical analyses in this study only included: five questions assessing salt-related knowledge (of which two had an additional open-ended sub-question), four questions in relation to attitudes towards salt, and four questions evaluating self-reported behaviour. Questions related to the presentation of salt/sodium content on the Nutrition Declaration Table were removed as they are no longer relevant. This study was conducted after the full implementation of the European Legislation (Regulation (EU) 1169/2011) which clearly specifies how the information on the salt content on food labels should be presented to consumers.

### 2.4. Score Calculations

In terms of knowledge assessment, a “knowledge score” was calculated by assigning a maximum of 5 points to each knowledge-related question. A total score of 5 points was allocated to participants who accurately identified the maximum daily salt intake recommended by experts, 3 points if they acknowledged the existence of recommendations but were unsure about the correct amount, and 0 points if they were unaware of the recommendations or believed that no recommendations existed. Regarding salt recommendations for children, 5 points were given if they indicated that children should consume less salt than adults, 3 points if they answered, “same amount as adults” and 0 points for “more salt” or “do not know”. In assessing the relationship between salt and sodium, 5 points were given for accurate knowledge of the relationship, 3 points for acknowledging the existence of a relationship without specifying it, and 0 points for lack of knowledge or denial of a relationship between salt and sodium. Regarding the association between high salt and serious health problems, 5 points were given for “Yes, there is an association” and 0 points for “No association” or “I do not know”. Lastly, regarding the association between high salt and in particular hypertension, 5 points were allocated for “high association”, 3 points for “possible association” and 0 points for “No association” or “I do not know”. Hence, salt-related knowledge scores ranged from 0 to 25 points.

In regard to salt-related behaviour, a score was calculated to assess the discretionary addition of salt. Five points were allocated if the participants “never” add salt during cooking, 3 points if they add salt “less than half of the meals”, 1 point if “more than half of the meals” and 0 if “always” or “I do not know/I do not cook”. Similarly, 5 points were allocated if “never” or “very rarely” add salt at the table, 3 points if this happens “less than half of the meals”, 1 point if “more than half of the meals” and 0 if “always”. The discretionary salt use score ranged from 0 to 10. The higher the score, the more consistent the knowledge and behaviour with national and international recommendations and policies.

### 2.5. Statistical Analyses

Categorical variables were presented as frequencies and percentages and continuous variables were given as mean with standard deviation. Univariate analyses were per-formed; the Kruskal-Wallis non-parametric test was used instead of ANOVA Test when the assumption of equal variance was violated. The multivariable mixed effect model was carried out to analyze the factors influencing the scores of salt-related knowledge and behavior by defining the random effects of the cohort. An overall cohort-adjusted model was calculated to assess the relation between salt consumption gr/day and knowledge, behavior scores, and such selected covariates (BMI; Sex and age in tertiles). Two models were created to investigate the same relationships as in the overall model, each stratified by a different random effect for cohorts: one for adults (Cohort I) and another for students (Cohort II). All descriptive statistics were analyzed using SPSS version 28.0, univariate and multivariable analyses were performed using R version 4.1.3. A *p*-value of <0.05 was considered statistically significant.

## 3. Results

### 3.1. Characteristics of Participants

**Figure 1:**
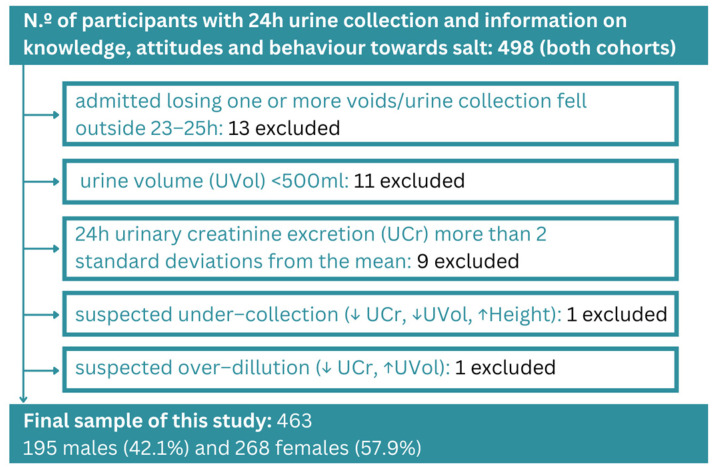
Flow chart of study participants.

The final sample comprised 463 participants from both cohorts: students from three universities/higher educational institutes and a general adult population (93% of the initial sample), of whom 268 (57.9%) were female. Figure 1 shows the quality control procedure carried out to ensure valid urine data in the analyses. The mean age was 35.1 years (SD ± 17.6), with a minimum and maximum of 18 and 76 years, respectively.

Table 1 provides the participant characteristics alongside salt intake distribution estimated from the 24-h urine collection.

### 3.2. Salt Intake Consumption

The mean salt intake estimated in this study, after combining data from two cohorts, was 9.54 g/day (10.94 g/day for males, 8.53 g/day for females). A small proportion of the samples (n = 61) (total: 13%, male: 6%, female: 19%) demonstrated compliance with the WHO recommendations of no more than 5 g of salt per day, with the majority of these individuals being female students (n = 43). In nearly half of the total sample (n = 218), daily salt intake ranged between 5.1 and 10 g per day (total: 47%, males: 44%, females: 49%), while in 40% of the total sample, salt intake was more than twice the WHO salt recommendations for adults (Table 1).

### 3.3. Sociodemographic Characteristics and Knowledge and Attitudes Scores

Only a minority of participants, specifically 55 individuals (11.9%), demonstrated accurate knowledge of the recommended salt intake for adults: 20 (10.3%) males and 35 (13.1%) females. In comparison to males, females were on average more knowledgeable presenting higher scores (34.17 ± 10.47 versus 31.57 ± 10.15; *p* = 0.009). The mean knowledge score was higher for people under 45 years old (33.37 ± 9.16 and 36.88 ± 9.89 compared to those who were 45 years old or older (28.91 ± 10.45) (*p* < 0.001). Considering the WHO standards for BMI categories, participants with a normal BMI (<25 kg/m^2^) had higher mean knowledge scores (34.57 ± 10.01) compared to those who were overweight (25.0–29.9 kg/m^2^) (31.0 ± 10.88) and obese (≥30.0 kg/m^2^) (31.43 ± 10.05), respectively (*p* = 0.002) (Table 2).

Regarding the behavior scores, a significant statistical difference was found between the age tertiles (*p* = 0.01). Participants between 22 to 44 years exhibited a higher mean behavior score than the age groups 45 years or older and under 21 years. There was no significant statistical difference between salt consumption and knowledge (*p* = 0.3) and behavior score (*p* = 0.06) (Table 2).

The findings of the mixed model analysis using salt consumption as a continuous dependent variable are presented in Table 3. According to the mixed model analysis, overall, there was a positive correlation between salt intake and BMI (β = 0.26; 95% CI: 0.17, 0.35; *p* < 0.001), suggesting that the higher their BMI, the more salt a person consumes. Additionally, compared to males, females demonstrated a significantly lower salt consumption (β = −2.1; 95% CI: −2.9, −1.4; *p* < 0.001). Regarding the salt consumption and knowledge (*p* = 0.562) and behavior scores (*p* = 0.210), no significant correlation was observed.

### 3.4. Attitude and Perception Regarding the Salt Content

Only 163 participants (35.6%) reported that salt reduction is “very important” for them, while 224 (48.9%) indicated “less importance” and 71 (15.5%) “no importance” regarding dietary salt intake (Table S1).

Approximately 45% of the participants held the belief that their salt intake was either lower or appropriate, while 24% perceived that their salt consumption was the recommended amount. Only 26.8% of the respondents thought that they consume more than the right amount and only 2.4% perceived their salt intake as “very high”.

The majority of participants (73.0%) stated that they made daily efforts to control their intake of salt intake. When they were asked to elaborate on these efforts, 32.2% indicated that they avoided or limited the consumption of processed foods, 42.5% reported that they did not add salt to their plates at the table, 16.4% reported that they used herbs or spices, and 24.0% of them desalted foods high in salt before eating them (Table S2). More than half of the participants (60.2%) agreed that the salt content of the food served in restaurants, taverns, and canteens was high or very high, but only a few (15.3%) claimed that one of the precautions they took to control their salt intake was to avoid eating out (Table S2).

Additionally, 35.2% of the participants claimed that they read the nutrition declaration table on food packaging more than half of the time or always, while approximately 25.5% of them stated that they never read the nutrition information on food labels (Table S1). Almost half of the participants (47.2%) indicated that they would like a clear, specific warning on high-salt foods (Table S2). Furthermore, 46.1% of the respondents also preferred an indication of the salt content of dishes on restaurant menus (Table S2). Among those who considered very important the reduction of salt in their diet, only 10.4% claimed that they always read, and 32.5% read more than half of the times the nutrition information on the food packaging (Table S1).

## 4. Discussion

This is the first study in Greece that investigated the association between salt intake based on a single 24 h urine collection and self-reported knowledge, attitude, and behaviour regarding salt. The primary observation of this study was that there was no correlation between salt intake and knowledge. Another important finding was that in nearly half of the total sample (n = 218), daily salt intake ranged between 5.1 and 10 gr/day, while in 40% of the total sample, salt intake was more than twice the WHO salt recommendations threshold for adults. Interestingly, only 26.8% of the respondents thought that they consume more than the right amount, only 2.4% perceived their salt intake as “very high”, and only 35.6% reported that salt reduction is very important for them.

In Europe and numerous high-income countries, a substantial proportion of sodium intake (70–80%) comes from the addition of salt during food manufacturing processes and food preparation in restaurants and mass catering establishments [1,20]. Our study suggests that current salt-related knowledge might be inadequate and insufficient to change behaviours and salt consumption. This highlights the need for prioritizing reformulation initiatives at the food industry and suppliers’ level, and establishing maximum salt content regulations, especially for processed foods that significantly contribute to overall dietary salt intake, which should come through in parallel with education programmes at a population level.

Food product improvement has been a priority in the European Union [21]. Recent studies have summarized the different strategies for reformulating foods to decrease salt content, highlighting the feasibility of reducing salt in processed items, such as bread and processed meats, without significantly affecting consumer acceptability [22,23]. However, in recent years there have been no revisions to the national Greek Food and Drink Code regarding the salt content of processed foods, primarily due to ongoing debate that prioritizes allocating resources towards consumer awareness initiatives. This study provides evidence supporting the need of accelerating food reformulation efforts, with the cereal-based products being of particular interest in the EU countries as showed in previous studies (e.g., [24]). A recent study [25] showed that reducing the salt content of bread, with or without dietary counseling, successfully reduced dietary salt intake without adversely affecting the dietary nutritional quality.

The results from our study are in accordance to previous studies in Greece [19,26], which showed that females appeared to have better salt-related knowledge than males. Surprisingly, this better knowledge did not translate into improved behavior regarding the use of discretionary salt. Students, university graduates, and people with lower BMI appeared to have more knowledge compared to other adults, and the same held true with respect to higher-educated individuals and people with lower BMI. However, only BMI had a positive correlation with salt intake. The positive correlation found between salt intake and BMI, albeit expected [27], is of great concern since obesity and high salt intake are two common risk factors strongly associated with non-communicable diseases, such as hypertension even in young adults [28].

Even though the majority of participants in this study consider making efforts on a daily basis to reduce salt intake, most do not seem to take concrete actions, which demonstrates the complexity of behaviour change and the difficulty of individuals to effectively control their salt intake. While various behavioural strategies to reduce salt (such as memory process strategy, gradual salt reduction strategy, and swap to low-salt food strategy) exist as described in a recent review by Nurmilah et al. [29], so far, such strategies have not been implemented at a population level in Greece. For example, most of the participants in this study reported that they did not limit the consumption of salt-rich processed foods and they did not buy low-salt food and low-sodium salt, although they were considering the reduction of salt consumption of importance. Therefore, since the intention to lower salt intake appears to exist, better labelling of the salt content of processed foods might be needed to facilitate and empower consumers to make healthier choices.

Less than half of the participants stated that they always or frequently read the nutrition information on the food labels. Interestingly, nearly half of the participants expressed the need to have clearer and more direct information regarding the food salt content, namely warnings on food packages (Front of Pack Nutrition Label, FOPNL) of foods high in salt, as well as salt content indications of the dishes in restaurants menus. Since the preference or understanding of different FOPNL schemes was not evaluated in this study, the preference of the study participants for warning symbol can only be viewed as participants’ preference to have a better labelling of nutrition information. So far, there is no consensus in the EU as to which FOP labelling system may be most effective.

During meal preparation, a minority of participants reported replacing salt with herbs or spices. Considering the trend observed in this study between discretionary salt use (behaviour score) and dietary salt intake, it is advisable to implement customized awareness initiatives aimed at promoting the substitution of salt with herbs, spices, and other sodium-free condiments in Greece. These awareness actions should be designed to target both household settings and mass catering establishments.

Although participants perceived meals provided in restaurants, taverns, and canteens as being high in salt content, the majority of participants stated they don’t refrain from dining out. In light of the increasing trend of consuming meals prepared outside of the home, especially through online food delivery services [30], which often provide energy-dense and high-in-salt meals, it is crucial to expedite awareness initiatives to reduce the salt content targeting those involved in mass catering, alongside food reformulation and effective regulations.

The main strength of this study was the use of 24-h urine sodium excretion to estimate salt intake. The association of salt intake with salt-related knowledge and self-reported behaviour was investigated for the first time in Greece, which could contribute together with other studies to the design of salt reduction policies and future actions. However, there are some limitations that should be acknowledged. Firstly, the sample was not nationally representative. The two cohorts included were from specific geographical areas of Greece (central and northern Greece) and included a large number of students. Consequently, our findings cannot be generalized to the whole country. Salt intake was estimated with the use of only one 24 h urine collection which is not expected to reflect the daily variation in salt intake and excretion in individual participants; however, it is the gold standard method for assessing salt intake at a population level. At present, there is no gold standard method for establishing a KAB score specifically pertaining to salt. Therefore, it is advisable that future research focuses on validating a salt-related KAB scoring system. Since the knowledge, attitude, and behaviour of participants were self-reported, the possibility of response bias due to social desirability cannot be ruled out. This study was based on a cross-sectional design and as a result, we were unable to detect causality between dietary knowledge/behaviour and salt intake assessed by 24 h sodium excretion. This may be due to lack of precision in characterizing an individual’s salt intake using a single 24 h urine collection because the intra-individual (day-to-day) variability in salt consumption often exceeds the between-individual (from person-to-person) variability in salt consumption [31]. Finally, we cannot exclude the possibility of other potential confounders which might have not been considered in this study.

## 5. Conclusions

The majority of participants had salt intakes well above the WHO recommendations, yet less than one-third considered salt reduction as highly important for their own health. Moreover, the participants’ limited salt-related knowledge suggested the potential for salt-related KAB improvement. Additionally, improving salt labelling on processed packaged food and the inclusion of salt content information for meals provided at restaurants and mass catering establishments, appeared to be preferred by many participants as a means to assist them in reducing their salt intake. These findings point to the need for food product reformulation by food industry and suppliers, alongside expediting measures and actions in this direction.

## Figures and Tables

**Table 1:** Characteristics of the participants and the salt intake distribution by cohort, sex, and in total.

	Total (n = 463)			Cohort I Adults (Excl. Students) (n = 200)		Students (Both Cohorts) (n = 263)	
	Male	Female	Total	Male	Female	Male	Female
	Mean ± SD	Mean ± SD	Mean ± SD	Mean ± SD	Mean ± SD	Mean ± SD	Mean ± SD
Age (years)	36.4 ± 17.7	34.2 ± 17.4	35.1 ± 17.6	52.3 ± 12.6	53.9 ± 11.1	21.7 ± 2.9	21.4 ± 1.8
Height (cm)	178.1 ± 7.2	164.2 ± 6.2	170.1 ± 9.6	176.9 ± 6.7	162.2 ± 6.5	179.3 ± 7.4	165.5 ± 5.7
Weight (kg)	82.1 ± 14.5	65.9 ± 12.7	72.8 ± 15.7	87.2 ± 13.3	73.4 ± 12.6	77.4 ± 13.9	62.1 ± 10.4
BMI (kg/m^2^)	25.8 ± 4.1	24.5 ± 4.9	25.1 ± 4.6	27.8 ± 3.7	27.9 ± 4.8	24.0 ± 3.6	22.3 ± 3.5
Waist Circumference (cm)	89.5 ± 13.4	76.9 ± 11.6	82.2 ± 13.9	97.4 ± 11.6	85.1 ± 12.4	82.5 ± 10.8	71.8 ± 7.3
	Median (IQR)	Median (IQR)	Median (IQR)	Median (IQR)	Median (IQR)	Median (IQR)	Median (IQR)
Daily Salt Intake (Tertiles)							
I (≤7.54)	6.01 (2.0–7.5)	5.3 (1.2–7.5)	5.5 (1.2–7.5)	6.0 (2.0–7.4)	5.8 (2.1–7.4)	5.8 (2.8–7.5)	4.9 (1.2–7.5)
II (7.57–10.63)	9.3 (7.6–10.6)	8.9 (7.6–10.6)	9.1 (7.6–10.6)	9.4 (7.7–10.6)	9.05 (7.6–10.6)	9.1 (7.6–10.4)	8.9 (7.7–10.6)
III (≥10.64)	13.5 (10.6–28.5)	12.8 (10.6–21.6)	13.3 (10.6–28.5)	13.6 (10.6–28.5)	13.4 (10.6–21.6)	12.8 (10.8–27.3)	12.3 (10.7–19.6)
	n (%)	n (%)	n (%)	n (%)	n (%)	n (%)	N (%)
Level of education (%)							
Secondary (non-students)	44 (23%)	65 (24%)	109 (23%)	44 (47%)	65 (61%)		
Students	101 (52%)	162 (61%)	263 (57%)			101 (100%)	162 (100%)
Tertiary (graduates)	50 (25%)	41 (15%)	91 (20%)	50 (53%)	41 (39%)		

**Table 2:** Associations between Selected Sociodemographic Characteristics and Knowledge and Attitudes Scores.

	Knowledge Score			Behavior Score (Discretionary Salt Use)		
	n	Mean ± SD	*p*-Value **	n	Mean ± SD	*p*-Value **
Sex			0.009			0.07
Male	190	31.57 ± 10.15		195	4.12 ± 2.31	
Female	262	34.17 ± 10.47		266	4.48 ± 2.37	
Age (tertiles)			<0.001			0.01
≤21 years	137	33.37 ± 9.16		138	3.94 ± 2.29	
22–44 years	162	36.88 ± 9.89		163	4.68 ± 2.53	
≥45 years	151	28.91 ± 10.45		158	4.31 ± 1.42	
Cohorts			<0.001			0.28
Adults (excl. students)	191	29.73 ± 10.91		199	4.20 ± 1.59	
All students	261	35.53 ± 9.31		262	4.42 ± 2.50	
Education			<0.001			0.46
Secondary	103	27.95 ± 11.25		108	4.12 ± 1.67	
Students	261	35.53 ± 9.31		262	4.42 ± 2.50	
Tertiary	88	31.81 ± 10.16		91	4.30 ± 1.50	
Body Mass Index (BMI)			0.002			0.1
<25	260	34.57 ± 10.01		262	4.51 ± 2.29	
25.0–29.9	128	31.0 ± 10.88		131	4.07 ± 2.06	
≥30.0	60	31.43 ± 10.05		64	4.10 ± 2.16	
Daily Salt Intake (Tertiles)			0.3			0.06
I (≤7.54)	153	34.07 ± 10.73		154	4.27 ± 2.18	
II (7.57–10.63)	149	32.66 ± 10.76		153	4.64 ± 2.23	
III (≥10.64)	150	32.48 ± 9.68		154	4.07 ± 2.02	

** ANOVA Test and/or Kruskal-Wallis non-parametric test.

**Table 3:** Mixed model analysis using salt consumption as a continuous dependent variable (overall and per cohort).

	Overall Cohort (463 Participants)			Cohort I Adults Excl. Students (191 Participants)			Students (Both Cohorts) (261 Participants)		
Sociodemographic	β	95% CI ^1^	*p*-Value ^2^	β	95% CI ^1^	*p*-Value	β	95% CI ^1^	*p*-Value
Body Mass Index (BMI)	0.26	0.17, 0.35	<0.001	0.19	0.05, 0.32	0.006	0.27	0.14, 0.40	<0.001
Sex			<0.001			0.002			<0.001
Male	—	—		—	—		—	—	
Female	−2.1	−2.9, −1.4		−1.8	−3.0, −0.70		−2.2	−3.1, −1.2	
Age (tertiles)			0.307			0.060			0.016
≤21 years	—	—		—	—		—	—	
22–44 years	−0.59	−1.5, 0.33		2.0	−3.9, 7.8		−1.2	−2.1, −0.22	
≥45 years	−0.74	−1.8, 0.31		0.16	−5.6, 6.0				
Knowledge score	0.01	−0.03, 0.05	0.562	0.05	−0.01, 0.10	0.098	−0.01	−0.06, 0.04	0.714
Behavior score (discretionary salt use)	−0.11	−0.28, 0.06	0.210	−0.31	−0.69, 0.07	0.105	−0.01	−0.20, 0.18	0.922

^1^ CI = Confidence Interval; ^2^ adjusted model for BMI, Sex and age in tertiles; *p* < 0.05 was statistically significant.

## Data Availability

The data presented in this study are available on reasonable request from the corresponding author.

## Supplementary Materials

The following supporting information can be downloaded at: https://www.mdpi.com/article/10.3390/nu15194114/s1, Questionnaire: Questionnaire on knowledge, attitude and behaviour with regard to salt; Table S1: Descriptive statistics of question 13, “Do you read the nutrition information on food packaging” and question 10, “How important is the reduction of salt in your diet” (in combination and separately); Table S2: Attitude and perception regarding the salt content.
