# Supplementary material for: Pertinence of Salt-Related Knowledge and Reported Behaviour on Salt Intake in Adults: A Cross-Sectional Study
Source: Nutrients. 2023 Sep 23;15(19):4114. doi: 10.3390/nu15194114 (PMC10574173; doi:10.3390/nu15194114)
Supplement: Supplementary file 1 [file nutrients-15-04114-s001.zip › nutrients-2608612-supplementary.pdf]

Questionnaire. Questionnaire on knowledge, attitude and behaviour with regard to salt

Gender: [ ] Male [ ] Female

Age:

Level of education: [ ] Secondary (Non-students) [ ] Students [ ] Tertiary (graduates)

1. Do you add salt when you cook?
   [ ] Always
   [ ] More than half of the meals
   [ ] Less than half of the meals
   [ ] Never
   [ ] Do not know/Do not cook
2. Do you add salt to your plate when you sit at the table?
   [ ] Always
   [ ] More than half of the meals
   [ ] Less than half of the meals
   [ ] Very Rarely
   [ ] Never
3. Is there a maximum daily amount of salt recommended for adults by experts?
   [ ] Yes [ ] No [ ] I do not know
   3a. If your answer is yes, which is the correct amount?
   [ ] Correct (5-6g) [ ] Incorrect [ ] No answer
4. Compared with adults, do you believe that children should consume…?
   [ ] More salt
   [ ] Same amount of salt
   [ ] Less salt
   [ ] Do not know
5. Is there a relationship between salt and sodium?
   [ ] Yes [ ] No [ ] I do not know
   5a. If yes, please specify:
   [ ] Correct [ ] Incorrect [ ] No answer
6. Do you believe that a diet high in salt could cause serious health problems?
   [ ] Yes [ ] No [ ] I do not know
   Q7 - A diet high in salt could cause <u>hypertension</u>
   [ ] High association [ ] Possible association [ ] No association [ ] Do not know
   Q7 - A diet high in salt could cause <u>obesity</u>
   [ ] High association [ ] Possible association [ ] No association [ ] Do not know
   Q7 - A diet high in salt could cause <u>osteoporosis</u>
   [ ] High association [ ] Possible association [ ] No association [ ] Do not know
   Q7 - A diet high in salt could cause stomach <u>cancer</u>
   [ ] High association [ ] Possible association [ ] No association [ ] Do not know
   Q7 - A diet high in salt could cause <u>kidney</u> stones
   [ ] High association [ ] Possible association [ ] No association [ ] Do not know
7. Q7 - A diet high in salt could cause high blood <u>cholesterol</u> levels
   [ ] High association [ ] Possible association [ ] No association [ ] Do not know

8. How much salt do you think you eat?
   [ ] Extremely high
   [ ] More than the right amount
   [ ] The right amount
   [ ] Less than the right amount
   [ ] Very little
   [ ] I do not know
9. What is the main source of salt in the diet of adults in Greece?
   [ ] Salt added on the plate at the table
   [ ] Salt added during cooking
   [ ] Bread
   [ ] Cheese
   [ ] Meat/processed meat
   [ ] I do not know
10. How important is the reduction of salt in your diet?
   [ ] Not important
   [ ] Less important
   [ ] Very important
11. Do you do something on a daily basis in order to control salt intake?
   [ ] yes [ ] no
   If yes, which of the following do you do?
   a. I avoid/limit consumption of processed foods [ ] yes [ ] no
   b. I check food labels [ ] yes [ ] no
   c. I do not add salt on my plate at the table [ ] yes [ ] no
   d. I buy low salt food [ ] yes [ ] no
   e. I buy low sodium salt [ ] yes [ ] no
   f. I do not add salt during cooking [ ] yes [ ] no
   g. I use herbs/spices instead of salt [ ] yes [ ] no
   h. I desalt foods (which are preserved by salt) before eating them [ ] yes [ ] no
   i. I avoid eating out [ ] yes [ ] no
   j. None of the above [ ] yes [ ] no
12. How do you assess the meals available in restaurants, taverns and canteens with regard to their salt content?
   [ ] Very little salt
   [ ] Little salt
   [ ] Right amount of salt
   [ ] High salt
   [ ] Very high in salt
13. Do you read the nutrition information on food packaging?
   [ ] Always
   [ ] More than half of the times
   [ ] Less than half of the times
   [ ] Never
14. Which of the following would you prefer to be mandatory?
   [ ] Salt labelling of all processed food
   [ ] Clear specific warning if a food is high on salt
   [ ] Salt content indication of dishes in restaurant menus
   [ ] None of the above

Table S1. Descriptive statistics of question 13, “Do you read the nutrition information on food packaging” and question 10, “How important is the reduction of salt in your diet” (in combination and separately)

			Importance to reduce salt in the diet			Total
			Not important	Less important	Very important	
Read the nutrition information on food packaging	Always	N	6	13	17	36
		%	8.5%	5.8%	10.4%	7.9%
	More than half of the times	N	19	53	53	125
		%	26.8%	23.7%	32.5%	27.3%
	Less than half of the times	N	25	100	55	180
		%	35.2%	44.6%	33.7%	39.3%
	Never	N	21	58	38	117
		%	29.6%	25.9%	23.3%	25.5%
Total		N	71	224	163	458
		%	15.5%	48.9%	35.6%	100.0%

Table S2. Attitude and perception regarding the salt content

		N	%	% without missing	% cumulative
Daily actions to control salt intake	Yes	338	73.0	73.0	73.0
	No	125	27.0	27.0	100.0
	Total	463	100.0	100.0	
If yes, which of the following do you do?	Avoid/limit consumption of processed foods	149	32.2	32.2	32.2
	Check food labels	59	12.7	12.8	12.8
	Don’t add salt to the plate at the table	197	42.5	42.5	42.5
	Buy low salt food	63	13.6	13.6	13.6
	Buy low sodium salt	31	6.7	6.7	6.7
	Don’t add salt during cooking	49	10.6	10.6	10.6
	Use herbs/spices instead of salt	76	16.4	16.4	16.4
	Desalt foods before eating them	111	24.0	24.0	24.0
	Avoid eating out	71	15.3	15.3	15.3
	None of the above	125	27.0	27.0	27.0
Personal perception of the salt content on the meals provided in	Very little salt	11	2.4	2.4	2.4
	Little salt	58	12.5	12.7	15.1
	Right amount of salt	113	24.4	24.7	39.7

restaurants, taverns and canteens	High salt	231	49.9	50.4	90.2
	Very high in salt	45	9.7	9.8	100.0
	Total	458	98.9	100.0	
Preference to be mandatory	Salt labelling of all processed food (incl. non-prepackaged)	11	2.4	2.4	2.4
	Clear specific warning if a food is high on salt	216	46.7	47.2	49.6
	Salt content indication of dishes in restaurant menus	211	45.6	46.1	95.6
	None of the above	20	4.3	4.4	100.0
	Total	458	98.9	100.0	
